# Localization of RNAs to the mitochondria—mechanisms and functions

**DOI:** 10.1261/rna.079999.124

**Published:** 2024-06

**Authors:** Surbhi Sharma, Furqan M. Fazal

**Affiliations:** Verna and Marrs McLean Department of Biochemistry and Molecular Pharmacology, Baylor College of Medicine, Houston, Texas 77030, USA; Therapeutic Innovation Center (THINC), Baylor College of Medicine, Houston, Texas 77030, USA

**Keywords:** imaging, mitochondrial biology, RNA subcellular localization, technologies, transcriptomics

## Abstract

The mammalian mitochondrial proteome comprises over 1000 proteins, with the majority translated from nuclear-encoded messenger RNAs (mRNAs). Mounting evidence suggests many of these mRNAs are localized to the outer mitochondrial membrane (OMM) in a pre- or cotranslational state. Upon reaching the mitochondrial surface, these mRNAs are locally translated to produce proteins that are cotranslationally imported into mitochondria. Here, we summarize various mechanisms cells use to localize RNAs, including transfer RNAs (tRNAs), to the OMM and recent technological advancements in the field to study these processes. While most early studies in the field were carried out in yeast, recent studies reveal RNA localization to the OMM and their regulation in higher organisms. Various factors regulate this localization process, including RNA sequence elements, RNA-binding proteins (RBPs), cytoskeletal motors, and translation machinery. In this review, we also highlight the role of RNA structures and modifications in mitochondrial RNA localization and discuss how these features can alter the binding properties of RNAs. Finally, in addition to RNAs related to mitochondrial function, RNAs involved in other cellular processes can also localize to the OMM, including those implicated in the innate immune response and piRNA biogenesis. As impairment of messenger RNA (mRNA) localization and regulation compromise mitochondrial function, future studies will undoubtedly expand our understanding of how RNAs localize to the OMM and investigate the consequences of their mislocalization in disorders, particularly neurodegenerative diseases, muscular dystrophies, and cancers.

## INTRODUCTION

The subcellular organization of the eukaryotic cell necessitates communication among organelles to maintain cellular functions. Coding RNAs transcribed in the nucleus act as messengers of genetic information, and their protein products are trafficked to various subcellular compartments in a regulated manner. Classic studies performed on Xenopus (Rebagliati et al. 1985; Weeks and Melton 1987) and Drosophila (St Johnston et al. 1991) oocytes first established that RNAs are distributed asymmetrically in the cell, with impairment to this localization pattern hindering embryo development. Further studies on specialized cells like neurons found that RNAs localize to distinct neuronal compartments. With advancements in imaging and sequencing approaches, RNA localization has been appreciated as a standard feature of eukaryotic cell organization.

Over the decades, numerous studies have revealed that both coding and noncoding RNAs exhibit specific subcellular localizations. This localization plays a pivotal role in ensuring the proper folding and availability of the associated protein products at precise cellular sites, thereby preventing misfolding and accumulation in unintended locations (Shakya et al. 2021). Moreover, beyond its impact on translation, localization may influence RNA folding, splicing, editing, and degradation processes. Notably, cells with extensive polarization, such as neurons and myocytes, are susceptible to disorders arising from mislocalization. In neurons, for example, nuclear-encoded RNAs can travel up to a meter or more to distal dendrites to influence synaptic function.

In this review, we examine RNA localization to the mitochondrial surface, where mitochondrial proteins are known to be translated (Kellems et al. 1974; Williams et al. 2014; Fazal et al. 2019; Uszczynska-Ratajczak et al. 2023). In addition, we highlight examples where mitochondria serve as a hub for vital RNA regulatory processes such as the innate immune response, piRNA biogenesis, and cytoskeletal-mediated RNA transport.

## MECHANISMS OF MITOCHONDRIAL RNA LOCALIZATION

The mitochondria are double-membrane organelles responsible for energy production. Mammalian mitochondria contain a 16.5 kb compact circular genome with 13 genes coding for proteins involved in oxidative phosphorylation. In addition, the mitochondrial genome encodes two ribosomal RNAs and 22 transfer RNAs (tRNAs). Mammalian mitochondria collectively harbor ∼1100 proteins (Calvo et al. 2016) across different cell types, of which only 13 are encoded by the mitochondrial genome. Thus, a large number of mitochondrial proteins are translated from nuclear-encoded mRNAs that are transported to mitochondria in a cotranslational or pretranslational state (Fazal et al. 2019). Upon reaching the outer mitochondrial membrane (OMM), these RNAs complete translation, and the resulting protein can be imported into the mitochondria by translocase complexes. The mitochondrial localization of these RNAs is likely precisely controlled by sequences (*cis*-elements) within the RNA and RNA-binding proteins (RBPs) (*trans*-factors) that interact with these sequences. Additionally, the repertoire and abundance of localized RNAs change in response to the cell's energy requirements, as demonstrated in yeast (Tsuboi et al. 2020).

The initial evidence for RNA localization on mitochondria came from studies on yeast spheroplasts that found ribosome-like structures in the mitochondrial fraction (Kellems et al. 1974; Ades and Butow 1980). The mitochondrial fraction showed amino acid incorporating ability, which was inhibited by cycloheximide, an inhibitor of protein synthesis. These observations hinted at the association of cytoplasmic ribosomes with mitochondria. Also, cycloheximide-mediated inhibition of this membrane association implies the presence of actively translating ribosomes at the mitochondria (Kellems et al. 1974). Electron microscopy-based observations confirmed this association and revealed the enrichment of bound ribosomes in regions of close contact between the outer and inner mitochondrial membrane (Kellems et al. 1975). Perhaps the proximity of the bound ribosome and of the two mitochondrial membranes mediates the efficient translocation of the translating polypeptides. The evidence for local translation at the mitochondria was further supported by the biochemical fractionation of mitochondria and their bound ribosomes by sucrose gradient centrifugation. The mitochondrial fraction isolated from yeast spheroplasts showed amino acid incorporation ability in an in vitro translation system (Ades and Butow 1980).

**FIGURE 1.**
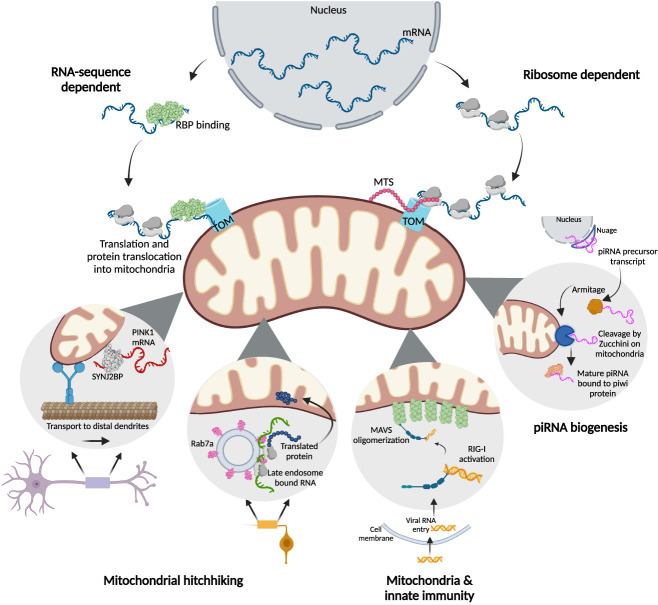
Modes of RNA localization to the mitochondrial membrane.

With the emergence of RNA-sequencing technologies, biochemical fractionation coupled with sequencing has revealed the localization of thousands of RNAs to the mitochondrial surface in yeast (Marc et al. 2002; Eliyahu et al. 2011). Since these initial findings, several studies have uncovered the mechanistic details of this localization process. Early studies established the role of sequence elements in the 3′ UTR for localization, with these sequence elements serving as recognition sites for RBPs. Later studies found that in addition to RNA sequence elements, translation is required for the localization of a subset of RNAs. With the availability of transcriptome-based approaches, more features governing RNA localization have since been uncovered. Below, we discuss the mechanisms modulating the RNA localization to the OMM (Fig. 1).

## RNA-SEQUENCE-DEPENDENT LOCALIZATION (*CIS*-ELEMENTS)

### Sequences in 3′ UTR and RBP binding

The OMM-localized RNAs can be classified into two broad categories based on their localization mechanisms: RNA-sequence-dependent and ribosome-dependent. The former mechanism requires sequences within the RNA to guide localization to the OMM, presumably mediated by the binding of RBPs. These RBPs typically bind sequence elements in the 3′-UTR region of mRNAs, escort them to OMM where they are translated, and the resulting peptide is subsequently imported into the mitochondria. In yeast, one well-studied RBP belongs to the PUF family of proteins, with initial studies establishing them to function as translational repressors (Wharton et al. 1998). Later studies identified the role of PUF proteins like Puf3p in mediating the localization of RNAs to OMM (Eliyahu et al. 2010; Gadir et al. 2011; Lapointe et al. 2018), with the deletion of Puf3p resulting in the mislocalization of many transcripts in yeast (Saint-Georges et al. 2008). Notably, mutations in the Puf3p binding motif of *bcs1* RNA affected its localization. These RBPs also suppress the translation of the bound RNAs in transit to the mitochondria to allow translation and efficient folding at the destination.

While the role of PUF proteins in mRNA localization is well established in yeast, recent discoveries are revealing similar mechanisms in higher organisms. In human cells, PUF homologs such as PUM1 and PUM2 have been recognized as translational repressors (Vessey et al. 2006; Uyhazi et al. 2020). However, their direct involvement in RNA localization is still under investigation. Interestingly, a comparable mechanism of OMM localization for nuclear-encoded mitochondrial RNAs has been observed in both flies and mammalian cells (Gehrke et al. 2015). This process is mediated by PUM and PINK1 proteins. Upon reaching the OMM, PUM-bound RNAs undergo derepression of translation, triggered by PINK1-mediated displacement of PUM repressors.

**FIGURE 2.**
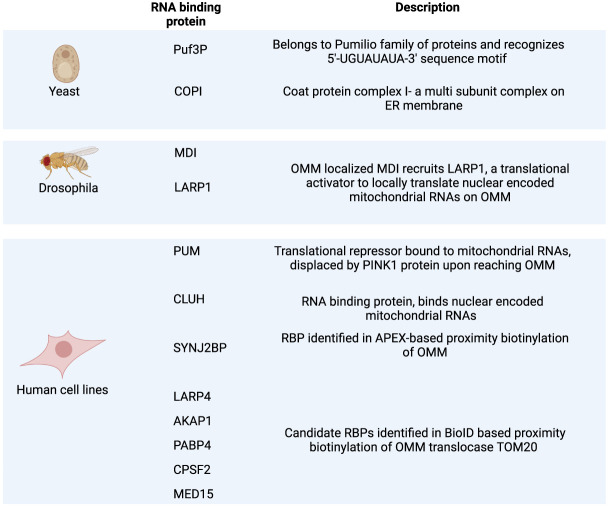
RNA-binding proteins (RBPs) are implicated in localizing RNAs to mitochondria.

Although many candidate RBPs that might control RNA localization have been identified (Fig. 2), it is still unclear what RBPs mediate localization in higher eukaryotes. For example, in mammalian cells, RBP CLUH preferentially binds nuclear mRNAs for mitochondrial proteins (Gao et al. 2014). The depletion of CLUH resulted in the reduced levels of protein products of the bound RNAs and mitochondrial morphology defects. The proximity labeling-based detection of protein–RNA complexes identified 28 OMM-localized RBPs in HEK293 cells, including the RBP SYNJ2B, which is thought to be involved in retaining ∼100 RNAs at OMM (Qin et al. 2021). Knockout of SYNJ2B abolished the localization of these mRNAs at the OMM and impaired the cellular stress response by compromising the function of oxidative phosphorylation complexes. Another recent study identified the proteins in proximity to TOM20 (OMM translocase) in human cells and found an overall enrichment of RBPs near TOM20, specifically proteins like AKAP1, LARP4, MED15, and CPSF2, thereby providing a candidate list of RBPs that might be involved in guiding mRNAs to the OMM (Meurant et al. 2023). Thus, although many RBPs are known to interact with nuclear-encoded mitochondrial transcripts, further studies are required to show that these RBPs play a role in RNA localization.

### Open reading frame sequences

Sequence elements within the open reading frame (ORF) can also be crucial in determining RNA localization. For instance, in yeast, mitochondrial dynamics undergo significant changes during the transition from fermentation to a respiratory mode of energy production (Egner et al. 2002). This transition has a subsequent impact on RNA localization. As cells switch to the respiratory mode, the volume of the mitochondria increases, thereby facilitating the conditional localization of specific RNAs, such as *atp3* and *tom22*. The larger mitochondrial volume in changing conditions elevates the likelihood that these RNAs will encounter the mitochondrial surface, as opposed to RNAs that are constitutively localized, such as *tim50*. It is also noteworthy that the ORF of *tim50* contains a sequence that encodes polyproline residues. This sequence causes ribosome stalling during translation, thus facilitating the recognition of the mitochondrial targeting sequence (MTS) and constitutive localization of *tim50* RNA on mitochondria (Tsuboi et al. 2020; Arceo et al. 2022).

### Other sequence elements

With the availability of transcriptome data based on proximity labeling of RNAs, it is now possible to derive sequence features contributing to RNA localization to the OMM. Machine learning-based predictions of the APEX-seq-based proximity biotinylation data set have unveiled the significance of several factors in OMM localization, including UTRs, poly(A) tail length, and short sequence motifs (Fazal et al. 2019). Notably, RNA-sequence-dependent transcripts exhibit shorter poly(A) tails and 3′ UTRs compared to RNAs relying on translation-dependent localization (i.e., ribosome-dependent RNAs). A recent study supported some of these findings by identifying mitochondria-bound RNAs derived from the larval stage of vertebrate zebrafish (Uszczynska-Ratajczak et al. 2023). The comparison of 18 zebrafish orthologues derived from the mitochondria-bound fraction through OMM proximity labeling of human cells revealed 17 out of 18 RNAs common in both (Fazal et al. 2019). They also found that ribosome-dependent RNAs coding for mitochondrial proteins contained longer ORFs and 3′ UTRs, further corroborating the APEX-seq findings in HEK293 cells (Fazal et al. 2019).

## RIBOSOME-DEPENDENT LOCALIZATION

The ribosome-dependent RNAs require the translation of the N-terminal peptide sequence, the MTS, for their localization to the OMM. These RNAs are likely transported cotranslationally after the MTS has been translated, but while the translating ribosome is still bound to the mRNA (Wang et al. 2016; Fazal et al. 2019). Once the MTS has been translated, it can be recognized by the translocase complexes on the OMM. This recognition allows the translating protein to be imported into the mitochondria. In yeast, for example, the localization of *atp2* mRNA was found to be dependent on the translation of the MTS. The introduction of the premature stop codon in the *atp2* sequence, which detaches the ribosomes from RNA, caused loss of localization (Garcia et al. 2010). The localization of ribosome-dependent RNAs was affected by factors that detach ribosomes from RNA, as observed by Saint-Georges et al. (2008); the addition of the translation-inhibitor puromycin abolished the localization of over 200 RNAs in yeast.

In human cells, thousands of RNAs accumulate at OMM for local translation. Proximity-labeling-based RNA sequencing (Fazal et al. 2019) revealed the enrichment of nuclear mRNAs coding for mitochondrial proteins at OMM. Furthermore, the localization of these RNAs was affected in the presence of various translational inhibitors. Cycloheximide, which prevents the elongation of translating ribosomes, typically enhanced the localization of ribosome-dependent transcripts, likely by enhanced localization of RNAs bound to stalled ribosomes that had synthesized a nascent peptide containing an MTS sequence. In contrast to cycloheximide, puromycin caused the detachment of translating ribosomes from the RNAs and typically abolished the localization of ribosome-dependent RNAs but not of the RNA-dependent ones. Localization of these RNA-sequence-dependent transcripts therefore occurs independent of translation, and does not require ribosome recognition of the translating N-terminal leader peptide.

Previously, studies in yeast had shown that a number of mitochondria-localized proteins, such as mitochondrial ribosomal proteins, contain internal targeting sequences that assist their import into mitochondria. Whether the RNAs coding for such proteins localize to OMM has not been well studied (Bykov et al. 2022). A recent study in human cells showed many of the RNAs coding for such mitochondrial ribosomal proteins localize to the OMM in a translation-independent manner (Fazal et al. 2019).

## OTHER MECHANISMS OF RNA LOCALIZATION TO AND RETENTION AT THE OMM

### RNA localization as part of cytoskeletal-based transport of mitochondria

Cytoskeleton-mediated transport is essential to orchestrate the RNA delivery to subcellular locations, and its significance becomes particularly evident in specialized cell types like neurons, where RNAs originating in the cell body undergo translation at distant locales such as axon terminals. Organelles like mitochondria and early endosomes use cytoskeletal microtubule networks as cellular highways to reach their destination, assisted by motor proteins such as dynein and kinesins. Several RNAs hitchhike on these organelles and take advantage of cytoskeletal-mediated transport to get to far locations. Examples of such hitchhiking have been observed in the axons of primary motor neurons derived from embryonic mice where *COX7C* mRNA colocalizes with mitochondria (Cohen et al. 2022). Further, live imaging unveiled the cotransport of *COX7C* mRNA with mitochondria along axonal processes, which was dependent on the MTS and ORF elements of the mRNA. Another recent study identified the cotransport of *PINK1* mRNA with mitochondria to distal neurites where local translation of the short-lived PINK1 protein assists in removing damaged mitochondria. The mitochondrial membrane protein SYNJ2B and its interacting partner SYNJ2 tether the *PINK1* mRNA via the RNA-binding domain of SYNJ2 and mediate its transport (Harbauer et al. 2022).

In addition to being localized on the mitochondrial surface, many RNAs encoding mitochondrial proteins are translated in the vicinity of mitochondria. Retinal ganglion cells (RGCs), responsible for transmitting visual signals over long distances, rely on the localized translation of RNAs within their axons. The FISH-based detection of *LB2* mRNAs in RGC axons and proteomic profiling of its newly synthesized protein validated its local translation (Yoon et al. 2012). In line with these observations, a study (Cioni et al. 2019) found that late endosomes carrying *LB2* mRNAs often dock at mitochondria in RGC axons. The proximity of endosomes and mitochondria ensures the delivery of translated LB2 protein to the mitochondria. Mutations affecting this process lead to mitochondrial aberrations and compromised neuronal function.

These studies have established the cotransport and local translation of RNAs tethered to or in proximity to the mitochondrial membrane and opened up new avenues for future research. For instance, the mechanisms behind the selective binding of RNAs coding for mitochondrial proteins and their tethering to the endosomal membrane are poorly understood. Recent studies are beginning to delve into these mechanisms by revealing the involvement of a multimeric Rab5-FERRY complex on endocytic vesicles, which plays a crucial role in binding RNAs encoding mitochondrial proteins (Schuhmacher et al. 2023). The cryo-EM structure of the FERRY complex identified the novel role of a coiled-coil domain of the FERRY subunit in RNA binding (Quentin et al. 2023).

Another instance of RNA hitchhiking was observed on lysosomes. Using proximity proteomics labeling, the ANXA11 protein was identified as a molecular tether that binds both RNA granules and the lysosomal membrane, enabling the transport of RNA granules along axonal lengths (Liao et al. 2019). Much like the interactions observed between endosomes and mitochondria, it remains unclear whether there exists mitochondrial proximity to lysosomes and if the eventual import of protein products derived from RNAs localized on lysosomes occurs.

The APEX-seq-based profiling of OMM transcriptome in the presence of nocodazole, which disrupts microtubule networks, revealed the loss of localization of the majority of OMM resident RNAs in HEK293 cells (Fazal et al. 2019). These observations suggest that microtubule-based transport might not be exclusive to specialized cells like neurons but a general phenomenon governing RNA localization across various cell types. Exploring how these RNAs tether to cytoskeletal networks and how the cross talk between organelles like endosomes and mitochondria is mediated are exciting topics to study in the future.

### Mitochondria and innate immunity

The OMM serves as an anchoring platform to host immune surveillance pathways. Upon infection by RNA viruses, RNA sensors like Retinoic acid-inducible gene-I (RIG-I) (Jiang et al. 2011; Kowalinski et al. 2011) and Melanoma differentiation-associated gene 5 (MDA-5) (Kato et al. 2008) recognize the viral RNA in the host cytoplasm. RIG-I primarily recognizes the 5′ triphosphate moiety of short double-stranded RNAs (dsRNAs) (Baum et al. 2010), whereas MDA-5 recognizes long dsRNAs. The binding of viral RNAs to these sensors induces a conformational change followed by oligomerization on the RNA. The RNA-bound oligomers of RIG-I (Thoresen et al. 2023) or MDA-5 then interact with mitochondrial antiviral-signaling protein (MAVS) on the mitochondrial surface. The N-terminal domain of RIG-I, MDA-5, and MAVS contains a caspase-recruitment domain (CARD), and the binding of the CARD domains of RIG-I and MDA-5 to the CARD domain of MAVS induces the oligomerization of MAVS. MAVS oligomerization leads to a cascade of events that activates transcription factors like NF-κB and IRF-3/7, culminating in the transcription and release of interferons (Seth et al. 2005; El Maadidi et al. 2014; Bender et al. 2015).

In addition to foreign RNAs, aberrant cellular RNAs can activate MAVS signaling. For example, cancer cells are well known to harbor a diverse array of aberrant RNA transcripts resulting from missplicing (Venables 2004; Kahles et al. 2018). Such missplicing overwhelms the spliceosome complex that processes these transcripts, thereby making these cells sensitive to interventions targeting the spliceosome. In such cases, inhibition of the spliceosome complex can be an effective approach to target tumor cells selectively. Treating breast cancer cells with small molecule modulators of the spliceosome leads to an accumulation of dsRNAs that results in the activation of the cellular antiviral response in these cells (Bowling et al. 2021). Sensing dsRNAs by cellular RNA sensors resulted in an aggregation of MAVS. It is thought that similar to viral RNAs, these misspliced RNAs with bound RNA sensors are also recruited to MAVS to activate an immune response, thus increasing the repertoire of RNAs that can localize to the OMM under different conditions. A recent report (Gokhale et al. 2023) showing that the OMM-localized MAVS directly interacts with 3′ UTRs of cellular RNAs through its intrinsically disordered domain reveals additional RNA-regulation functions by MAVS.

### piRNA biogenesis

The germline cells of metazoans contain nonmembranous perinuclear structures (nuage), which serve as a site for the biogenesis of piRNAs. piRNAs are small RNAs (∼30 nt) that mediate the silencing of transposons in germ cells, thereby ensuring fertility. Electron microscopy-based observations of mouse cells have revealed that nuage forms close contacts with mitochondria, hinting toward the role of mitochondria in piRNA biogenesis (Aravin et al. 2009). Ectopic expression of MitoPLD, an endonuclease of the piRNA biogenesis pathway, showed colocalization with a mitochondrial marker (Huang et al. 2011; Watanabe et al. 2011). The biochemical fractionation of mouse testicular cells further confirmed its expression in the mitochondrial fraction (Watanabe et al. 2011). The depletion of MitoPLD in mouse models caused the meiotic arrest and male infertility. Similar findings have been observed in ovarian soma cells of Drosophila, in which Zucchini, the MitoPLD homolog, colocalized with mitochondria (Saito et al. 2010). In Drosophila germ cells, the piRNA production occurs in two phases in physically separate locations, nuage and OMM. The piRNA precursors generated in nuage move to the mitochondrial surface for further processing. Ge et al. (2019) have shown that the RNA-binding ATPase protein, Armitage, localizes on the mitochondrial surface and shuttles piRNA precursors from perinuclear nuage to mitochondria in Drosophila. Thus, key steps in piRNA regulation likely occur on the mitochondrial surface, and further studies will undoubtedly reveal the extent and timing of such regulation at the OMM.

### RNA import into mitochondria

Although RNA localization to the OMM is widespread, a few RNAs are known to be imported into mitochondria. The evidence for the import of nuclear-encoded RNAs into mitochondria came from classic studies conducted in *Tetrahymena*, whose mitochondria contains tRNAs that originate from either the nuclear or mitochondrial genome (Chiu et al. 1975; Suyama 1986). Later studies on *Trypanosoma brucei*, a protozoan whose mitochondria does not encode tRNAs and solely rely on the import of nuclear-genome-encoded tRNAs, further confirmed tRNA import into mitochondria (Schneider et al. 1994; Hauser and Schneider 1995). In yeast and higher eukaryotes, the import of tRNAs coding for lysine has been observed (Tarassov et al. 1995). Although the mechanism of this import remains understudied, studies have shown that in yeast, the import requires the protein import channels and electrochemical potential of the OMM (Kolesnikova et al. 2000), while additional studies identified the role of enolase (Brandina et al. 2006) and lysyl tRNA synthetase (Kamenski et al. 2010), which act as carriers during this process. Subsequent studies demonstrated the import of glutamine tRNA in isolated mitochondria derived from yeast (Rinehart et al. 2005), rat, and human cells (Rubio et al. 2008). The in vitro transport of this tRNA was dependent on ATP and a functional ATPase. In addition, the import was abolished when isolated mitochondria were treated with digitonin that disrupts only the OMM, thereby hinting at the role of intact mitochondria and perhaps its protein channels in the import process in higher eukaryotes (Rubio et al. 2008). In addition to tRNAs, nuclear-encoded 5S rRNA is also reported in the mitochondrial matrix (Magalhães et al. 1998), while later studies identified RNA structural elements in the 5S rRNA that mediate its import into mitochondria (Smirnov et al. 2008). A recent study investigated the role of long noncoding RNAs (lncRNAs) in ulcerative colitis, uncovering an association between decreased levels of the nuclear-encoded lncRNA HOXA11os and increased disease severity. HOXA11os RNA colocalized with complex I of the electron transport chain, and reduction in abundance compromised oxidative phosphorylation, thereby contributing to disease severity (Shmuel-Galia et al. 2023). Given the limited studies available, whether other RNAs are imported into mitochondria or not remains a subject for future research.

## TECHNIQUES TO STUDY RNA LOCALIZATION

### Imaging

**FIGURE 3.**
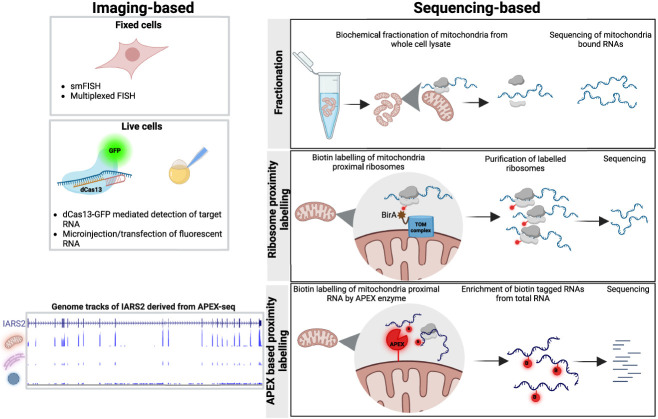
Toolbox to study RNA subcellular localization.

The toolbox to study subcellular localization is continuously evolving and can be broadly categorized into imaging-based and sequencing-based approaches (Fig. 3). Imaging-based approaches have a long history in RNA localization studies. For instance, a classic study by Singer and Ward (1982) used a biotin-dUTP probe to detect actin mRNA in muscle cells using an avidin-dye conjugate. Since then, many advancements in FISH protocols have been made to enhance the signal. In one approach, multiple fluorescent probes bind to the RNA body forming a tiling array, thereby enhancing the signal quality and specificity (Femino et al. 1998; Raj et al. 2008; Trcek et al. 2017). Later approaches like RNAscope (Wang et al. 2012) use secondary and tertiary probes that bind the primary probe hybridized to the RNA to improve the fluorescence. A recent method called clampFISH uses click chemistry to ligate the primary probe to the target followed by the binding of fluorescent secondary and tertiary probes, significantly enhancing the fluorescence intensity (Rouhanifard et al. 2019). Recently developed RNA aptamers like SPINACH (Paige et al. 2011) and MANGO (Autour et al. 2018) have also been used to image cellular RNAs. These aptamers emit fluorescence by converting a nonfluorescent ligand to a fluorescent state upon RNA binding. Similar approaches continue to be developed to visualize RNAs of interest in cells. However, a general limitation of imaging-based approaches is that they need prior information about the sequence of RNA of interest, since specific probes need to be added to recognize and bind to the RNA(s).

With continued advancements in imaging tools and analysis algorithms, the visualization of multiple RNAs has become feasible in the past decade. One of the pioneering approaches for multiplexed RNA imaging used five barcodes with distinct spectral properties to image 10 unique RNAs in cells (Levsky et al. 2002). On the same line, sequential hybridization of fluorescent probes and two rounds of hybridization visualized 12 RNAs in yeast (Lubeck and Cai 2012). The highly advanced versions of the above approaches, such as MERFISH (Xia et al. 2019) and seqFISH (Eng et al. 2019), can detect thousands of transcripts by sequential hybridization of FISH probes in multiple rounds to reveal the location of RNAs in cells. MERFISH coupled organelle imaging revealed the subcellular location of RNAs in the ER and nucleus. Further, MERFISH coupled with expansion microscopy, which expands the fixed cell and clears proteins and lipids, has increased the number of detectable RNAs by reducing molecular crowding and improving spectral decoding (Wang et al. 2018).

While the aforementioned methods detect RNAs in fixed cells, currently imaging RNAs in live cells is limited to only a few transcripts at a time. Initial approaches performed microinjection of fluorescent RNAs into Drosophila oocytes to image the localization and dynamics of injected RNA (Glotzer et al. 1997; Cha et al. 2001; Wilkie and Davis 2001). The MS2 system in yeast leveraged GFP fused to RNA-binding phage protein MS2 and a reporter RNA with stem–loop binding sites for MS2 (Bertrand et al. 1998). The MS2 system showed the role of *ash1* 3′ UTR in mediating localization to the bud region in yeast. Since then, improvements have been made in the MS2 reporter to increase the signal-to-noise ratio (Wu et al. 2015; Park et al. 2020). Furthermore, with the advent of CRISPR–Cas9-based genome editing, a number of Cas9 variants have been discovered and applied to image RNAs. For example, a pioneering study (Nelles et al. 2016) used the PAM recognition feature of Cas9 to image RNAs. Briefly, the study used a PAM sequence-containing oligo to bind to target RNA followed by visualization of RNA-oligo hybrid using GFP-tagged Cas9 lacking catalytic activity. Using this approach, the investigators showed the localization of *ACTB*, *TFRC*, or *CCNA2* mRNAs in stress granules. Recently, catalytically dead versions of RNA-cleaving Cas proteins like Cas13a and Cas13b tagged with GFP have also been used to locate RNAs and even track their dynamics (Abudayyeh et al. 2017; Yang et al. 2019). Live cell imaging, though limited to a few RNAs, facilitates careful dissection of the dynamics of the RNA localization process.

### Sequencing

The sequencing-based approaches reveal the subcellular locale of RNAs in a high-throughput manner. The biochemical fractionation of organelles followed by sequencing has identified the RNA population of the nucleus, mitochondria, and even membrane-less organelles like nucleolus (Tilgner et al. 2012) and stress granules (Khong et al. 2017). While these fractionation protocols have advanced our understanding of the organelle transcriptome, they are less suited for profiling RNA populations in membrane-less organelles like the OMM, nuclear lamina, and stress granules.

Initial efforts to profile the RNA population at these locations used proximity labeling to biotinylate ribosomes associated with ER (Jan et al. 2014; Costa et al. 2018) and mitochondrial surface (Jan et al. 2014; Vardi-Oknin and Arava 2019). This protocol was followed by the isolation of biotin-tagged ribosomes and their bound RNA followed by sequencing, which revealed the RNA population enriched at these sites.

Recent developments in RNA proximity labeling-based approaches, such as APEX-seq (Fazal et al. 2019; Padrón et al. 2019; Barutcu et al. 2022) and Halo-seq (Lo et al. 2022), have expanded our understanding of RNA organization within cells and broadened the scope of subcellular locations whose transcriptomes can be explored. APEX, an ascorbate peroxidase, can be targeted to various organelles by tagging it with proteins that reside at the site of interest or by adding short localization signals. Using this approach, Fazal et al. profiled the transcriptome from nine subcellular locations, including the mitochondrial surface and matrix, uncovering previously unknown resident RNAs. Moreover, the addition of translational inhibitors like cycloheximide and puromycin, followed by APEX-seq revealed RNAs that depend on or are independent of translation for localization to the mitochondrial surface. Other sequencing-based approaches continue to be developed (Medina-Munoz et al. 2020; Pani et al. 2023). One such approach, for example, tags a polyuridine polymerase to mitochondrial and ER membrane-localized proteins in yeast, which adds a stretch of poly(U) to RNAs in proximity, which can then be sequenced to identify uridylated RNAs (Medina-Munoz et al. 2020).

## FUTURE PERSPECTIVES

The discovery of mitochondria-bound cytoplasmic ribosomes, followed by various studies on examining OMM-proximal RNAs, has led to the identification of regulatory mechanisms of RNA localization on OMM. Nonetheless, many aspects of the RNA localization process remain unexplored. In particular, although a few RBPs that mediate the RNA localization process have been identified in yeast, the corresponding RBPs in higher organisms remain unknown. However, we have a candidate list of RBPs like AKAP1, LARP4, MED15, and CPSF2 that could mediate localization, as ascertained from proximity labeling of proteins near TOM complexes. Future studies will undoubtedly seek to establish the direct role of these and other RBPs in mediating RNA localization to the OMM, thereby providing new insights into how the mammalian mitochondria are regulated. Concomitantly, the identification of short sequence motifs or zip codes responsible for RNA localization is possible now due to recent advancements in experimental and analysis tools for MPRA screens. Massively parallel reporter assays (MPRAs) entail the introduction of many (typically thousands to tens of thousands) of short sequences (up to ∼100–200 bp) in a reporter plasmid, introduction of the plasmid into cells, and subsequent identification of these motifs in transcribed RNAs at the location of interest by sequencing. MPRA screens have been used extensively to interrogate zip codes of nuclear lncRNAs (Shukla et al. 2018), cytoplasmic circRNAs (Ron and Ulitsky 2022), and dendrite-enriched mRNAs (Mendonsa et al. 2023). Future studies aimed at MPRA screens of OMM-localized RNAs will lead to the identification of not only zip code elements but also their potential RBP partners.

In addition to sequence motifs, other features of RNA such as RNA modifications, RNA structure, and the poly(A) tail may mediate the localization of transcripts. RNA modifications are regulatory marks added posttranscriptionally, further increasing the complexity of the transcriptome and expanding the scope of *cis*-regulatory features. For instance, m^6^A, methylation of adenine base at the N6 position, has been detected in almost one-third of the mammalian transcriptome (Dominissini et al. 2012; Meyer et al. 2012). These m^6^A sites tend to be enriched in the 3′-UTR region of mRNAs and can potentially influence RNA localization by modulating the RNA secondary structure or the binding of RBPs. Differential RNA editing is associated with various neurological disorders (Li and Church 2013). For instance, a recent study found reduced levels of adenosine to inosine modifications in brain tissues obtained from schizophrenia patients (Choudhury et al. 2023). Remarkably, these hypo-edited sites were enriched in the 3′ UTR of RNAs associated with mitochondrial processes. Investigating the impact of these modifications on RNA localization to the OMM and mitochondrial function will shed light on the effect of editing defects. In addition to the highly abundant modifications like m^6^A and inosine, the transcriptome encompasses more than a hundred types of chemical modifications (Roundtree et al. 2017). The next step in our understanding of epitranscriptome-mediated regulation of RNA localization on the OMM is to explore how these diverse modifications affect the localization of target RNAs and, thereby, organellar function.

Additionally, the secondary structure of RNAs could influence RBP binding, which in turn could influence the localization of RNAs transiting to OMM and their translation. With recent advancements in structure-probing techniques (Siegfried et al. 2014; Spitale et al. 2015; Sun et al. 2019), a structure-probing method coupled with proximity labeling like APEX-seq could uncover the structural landscape of RNA as it travels from the nucleus to OMM.

Central to understanding mitochondrial function is elucidating how the nuclear and mitochondrial genomes coordinate and how RNA localization to OMM differs among cell types. Studies have shown that mitochondrial proteins encoded by the nuclear genome are differentially expressed among mouse tissues (Pagliarini et al. 2008). Another study (Fecher et al. 2019) has demonstrated that mitochondria derived from three brain cell types contain distinct proteomes. In particular, astrocyte-derived mitochondria metabolize fatty acids more efficiently than those of neurons, as reflected in their mitochondrial proteomes. It remains unknown whether these variations in the mitochondrial proteome modify the OMM-localized transcriptome, and further investigation is required.

Several mitochondrial diseases arise from mutations in nuclear-encoded mitochondrial proteins that show tissue-specific expression, resulting in organ-specific dysfunctions. For instance, Leigh syndrome is characterized by mutations in genes from both the mitochondrial and nuclear genomes, with infants born with this disorder showing neurological dysfunction. Apart from disease-related changes, mitochondria exhibit dynamic behavior in response to various stressors encountered on a daily basis. These stressors range from reactive oxygen species, metabolic overload, infection, to the buildup of misfolded proteins. Studies in the past years have revealed that in response to stresses like amino acid overload and aging, the OMM forms distinct compartments known as mitochondria-derived compartments (MDCs) (Hughes et al. 2016; Schuler et al. 2021). These MDCs containing select OMM proteins such as TOM70 and SLC25A, can take part in adaptative responses to amino acid overload. In yeast, aging mitochondria form MDCs that are destined for degradation in lysosomes. The formation of MDCs renders such cells capable of mitigating the effect of these stresses. In response to infection by *Toxoplasma* parasites that attach to the OMM of the host cells, mitochondria shed vesicle-like structures containing proteins of the OMM, as part of the cellular defense to restrict the parasite growth (Li et al. 2022). Thus, dissecting the contribution of RNA localization to mitochondrial function in various cellular contexts will be crucial in understanding the RNA localization-mediated regulation of mitochondrial biology.
